# Hierarchical graph contrastive learning of local and global presentation for multimodal sentiment analysis

**DOI:** 10.1038/s41598-024-54872-6

**Published:** 2024-03-04

**Authors:** Jun Du, Jianhang Jin, Jian Zhuang, Cheng Zhang

**Affiliations:** 1 School of Physics and Electronics, Shandong Normal University, Shandong, China; 2 School of Ethnology and Sociology, Yunnan University, Yunnan, China

**Keywords:** Computational science, Computer science, Information technology

## Abstract

Multi-modal sentiment analysis (MSA) aims to regress or classify the overall sentiment of utterances through acoustic, visual, and textual cues. However, most of the existing efforts have focused on developing the expressive ability of neural networks to learn the representation of multi-modal information within a single utterance, without considering the global co-occurrence characteristics of the dataset. To alleviate the above issue, in this paper, we propose a novel hierarchical graph contrastive learning framework for MSA, aiming to explore the local and global representations of a single utterance for multimodal sentiment extraction and the intricate relations between them. Specifically, regarding to each modality, we extract the discrete embedding representation of each modality, which includes the global co-occurrence features of each modality. Based on it, for each utterance, we build two graphs: local level graph and global level graph to account for the level-specific sentiment implications. Then, two graph contrastive learning strategies is adopted to explore the different potential presentations based on graph augmentations respectively. Furthermore, we design a cross-level comparative learning for learning local and global potential representations of complex relationships.

## Introduction

Multimodal data, such as textual, acoustic and visual information, has become an important means of communication for individuals and the public as social media has grown in prevalence. In this scenario, estimating human sentiment tendencies from multimodal data becomes increasingly important. Therefore, multi-modal sentiment analysis (MSA)^1–3^ on multimodal data has become a hot topic in multimedia content understanding (MCU) and natural language processing (NLP). Its have been widely used in industrial and academic communities, such as social media analysis^4^, dialogue systems^5^, e-commerce promotion^6^ and human–computer interaction^7^.

To effectively understand multimodal information, Early MSA work attempted to fuse the information from different modalities by tensor-based features fusion^8,9^ or attention-based features fusion^10,11^. Furthermore, some representation learning-based approaches^12,13^ aim to model the consistency and the variability between modalities for extracting the sentiment cues among modalities or consider both fusion and alignment of multimodal sequential data with a graph model^14,15^. Researchers have focused on graph neural networks and proposed hierarchical graph contrastive learning frameworks to explore the complex relationships of intra-modal and inter-modal representations for extraction^16^. They have also developed global and local fusion neural networks that aggregate global and local fusion features to analyze user emotions^17^. Additionally, they have used linguistic methods to extract sequential features from multimodal modeling and represented emotional associations through hidden Markov model^18^. Despite the promising progress made by current work, they generally focus on fusing multimodal representations via multimodal data within a single instance, which ignores single instance have specific global co-occurring characteristics. How to more effectively make use of the feature co-occurrences across instances and capture the global characteristics of the data remain a great challenge.

In this paper, we study how to capture the global characteristics of the multimodal data and explicitly model the global feature, enabling the highly correlated modal representations to be explicitly linked for learning the multimodal sentiment information. To reach this goal, we propose Hierarchical Graph Contrastive Learning (HGCL-LG), which constructs a network based on comparative learning to realize multiple levels of information exploration. Specifically, since discrete variational autoencoder (dVAE)^19^ can map different samples into a common discrete embedding space, we assume that this embedding space contains global information between samples. Therefore, we use dAVE to get the embedding space for each modal. On this basis, we construct local graph and global graph, and design three comparative learning: local graph contrastive learning, global graph contrastive learning and cross-level graph contrastive learning. By the three comparative learning methods, our model fully learns the sentiment features in local information and global information and the complex relationship between the two. In addition, we introduce an adaptive graph augmentation strategy, which can automatically node augmentation, as far as we know, this is the first time this strategy has been applied to an MSA task.

In brief, the contributions of our work can be summarized as follows:

- We approach the MSA task from a novel perspective, which explicitly models both global and local information to exploit the latent representations and sentiment relationships of global and local information.
- We designed a new hierarchical graph contrast learning (HGCL-LG) framework for extracting sentiment relations at the local level and the global level.
- In the graph contrast learning-based MAS task, we introduce an automatic graph augmentation strategy for exploring better multimodal graph structures.
- Performance evaluation on CMU-MOSI and CMU-MOSEI datasets shows the superiority and robustness of the proposed framework compared to several competitive baselines.

The remainder of this study is structured as follows. Section “Related works” mainly introduces two aspects of research: multimodal sentiment analysis and contrastive learning. Section “Methodology” provides a detailed description of the proposed HGCL-LG architecture and describes the training process of hierarchical graph contrastive learning. Section “Experiments” introduces the experimental setup, baseline model description, and conducts comparative experiments between HGCL-LG and baseline models, as well as ablation experiments and visualization of experimental results. Finally, Section “Conclusion” summarizes all the findings and draws conclusions.

## Related works

Multi-modal sentiment analysis has attracted extensive attention in the multimedia community in recent years^20,21^, because of the vivid and interesting information in multi-modal data, In the following, we mainly present the related works on the traditional MSA model without cross-instance information and our proposed approach.

### Multimodal sentiment analysis

The goal of MSA is to regress or classify the overall sentiment of an utterance via acoustic, visual, and textual cues. The models like TFN^8^ and LMF^9^ use tensor-based method to get joint representation for utterances. MSAF^10^ design a weighted cross-modal attention mechanism to explore cross-modality interactions. MAMN^11^ employs a multi-level attention map network to filter noise before multimodal fusion and capture the consistent and heterogeneous correlations among multi-granularity features for multimodal sentiment analysis.

Those methods have been applied to extract the features of Euclidean structure data with great success. The performance of those methods on non-Euclidean structure data like graph data is still unsatisfactory. Graph neural networks (GNN)^22^ is proposed to handle graph-structured data for capturing the interaction between nodes. Multimodal Graphs^15^ transform sequential learning problem into graph learning problem, which can effectively learn longer intra- and inter-modal temporal dependency. TGCN^23^ introduces graph convolutional network to obtain modality-specific semantic information, and the author devise a two-stage attention fusion network to fuse the feature at modality-specific level and cross-modal level.

The above methods have showed excellent performance in MSA. However, these models are employed to explore the relationship between multimodal information in a single instance, and the extra processing for cross-instance information does not exist. We propose a novel graph-based approach to learn the relationship of cross-instance.

### Contrastive learning

Our work also relates to contrastive learning. Contrastive learning (CL) is originally proposed as a self-supervised learning method for solving the lack of supervised signals^24,25^. CL often requires effective data augmentation as a foundation. MISA^13^ learns modality-invariant and modality -specific representation for each modality to improve the fusion process. MMCL^26^ has been proposed to capture intra-modality and inter-modality dynamics simultaneously. The combination with graph networks is another new application of contrastive learning^27,28^. The graph networks can model the association between nodes, and data augmentation on graph structures is feasible and operable. Common augmentation methods include additions and deletions of nodes or edges, masking of the representations of nodes or edges, etc., which usually cannot adapt to input data or preserve the original semantic structures well^29^. Therefore, to explore more appropriate graph structures, inspired by^29^, we apply the graph augmentations by automated deleting and masking nodes in graphs, and thus derive multifarious but similar graph structures with respect to the source.

## Methodology

**Figure 1:**
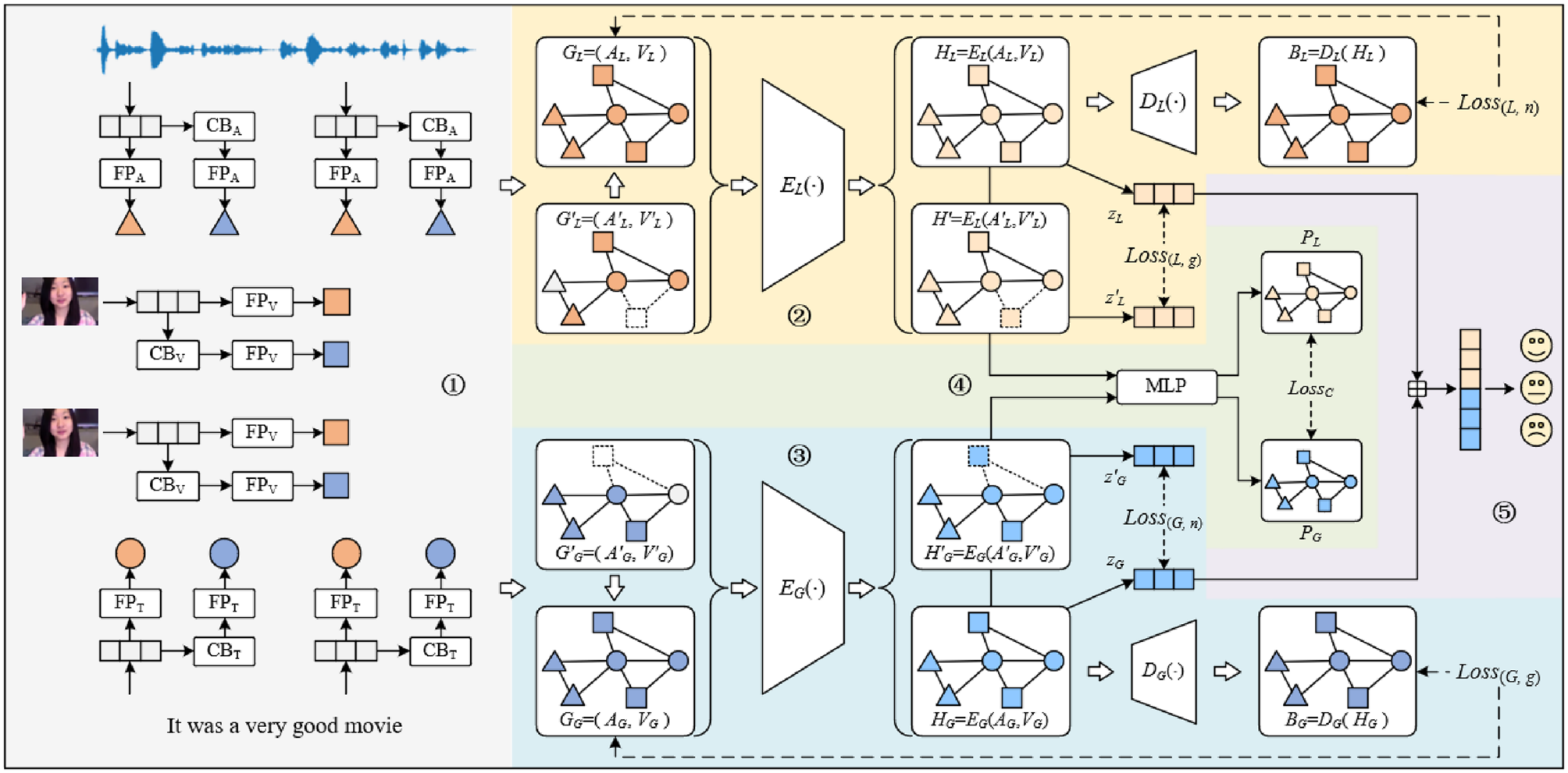
The overall architecture of our proposed HGCL-LG framework. The model consists of five main modules: ① Graph Construction, ② Local-Level Graph Contrastive Learning, ③ Global-Level Graph Contrastive Learning, ④ Cross-Level Graph Contrastive Learning and ⑤ Fusion and Sentiment Prediction.

In this section, we begin with our task formulations first. Then, we present our proposed HGCL-LG in detail. The architectures of our HGCL-LG are shown in Fig. 1. Finally, we describe the training process of hierarchical graph contrastive learning.

### Task setup

Formally, supposing there is a sample consisting of a text t and the corresponding image frames v and audio a from a video, multimodal sentiment analysis (MSA) aims to predict a sentiment score y, which is a constant from − 3.0 to 3.0, for each sample. In addition, according to the sentiment score y, we thus identify the sentiment polarity (i.e. positive if y > 0, neutral if y = 0 and negative if y < 0).

### Graph construction

This section describes how to construct the local and global graphs for each multimodal instance.

The raw multimodal sequence features are extracted directly from one utterance sample and do not consider the relations with other samples in the dataset, we define as local sequence features. In contrast, sequence features that consider the relationship between samples in a dataset are defined as global features.

#### Create codebook

dVAE can learn an embedding space from a dataset, and this embedding space includes the global co-occurrence features of the dataset.

We use acoustic modalities as an example to explain the process of creating codebook. First, given a raw acoustic sequence feature ${X}_{a}$, which can define as:

1 $$X_{a} = \left\{ {a_{i} } \right.\left| {i = } \right.1, \ldots ,\left. {T_{a} } \right\} \in {\mathbb{R}}^{{T_{a} \times d_{a} }}$$

where ai represents the i-th vector of sequence features. Ta is the sequence length and da is the representation vector dimension. Then, dVAE takes the acoustic sequence features of all samples in the training set as input to obtain the acoustic codebook ${CB}_{a}$:

2 $$CB_{a} = \left\{ {cb_{a}^{k} \left| {k = 1, \ldots ,k_{a} } \right.} \right\} \in {\mathbb{R}}^{{K_{a} \times d_{a} }}$$

where ${cb}_{a}^{k}$ denotes the *k*-th vector of acoustic codebook, and ${k}_{a}$ denotes the size of discreate space. Finally, following the same method, we get the textual codebook ${CB}_{t}$ and the visual codebook ${CB}_{y}$.

#### Building local graph

To leverage the intricate sentiment implications within local features, we construct a local multimodal diagram based on the original sequence features.

**Figure 2:**
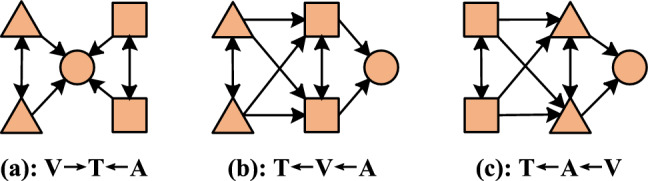
Three ways of edge construction, circles represent text nodes, triangles represent audio nodes, and squares represent video nodes.

*Node construction* As illustrated in Fig. 1, each modality’s input feature vectors are first passed through a modality-specific Feed-Forward-Network. This allows feature embeddings from different modalities to be transformed into the same dimension. Then, a positional embedding is added (separately for each modality) to each embedding to encode temporal information. The output of this operation becomes a node in the graph (Fig. 2).

**Figure 3:**
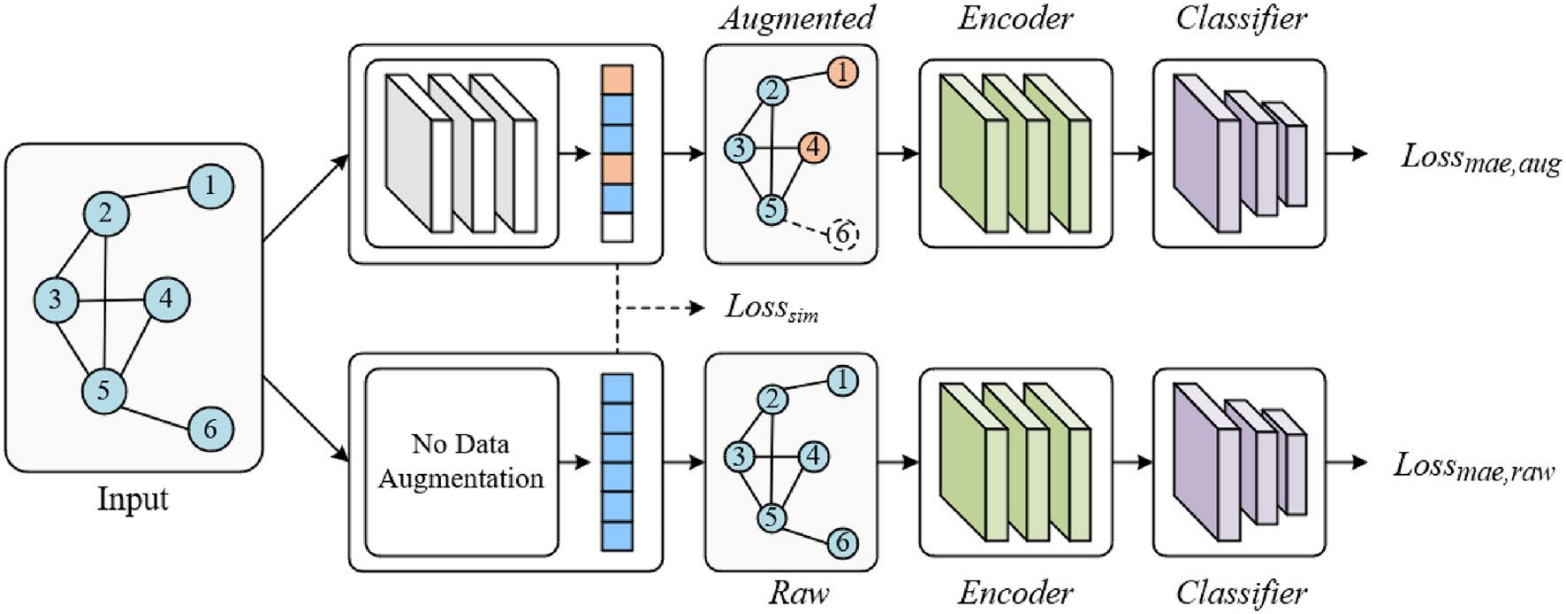
The architecture of automatic graph data augmentation strategy. The GNN layers embed the original graph to generate a distribution for each node. The augmentation choice of each node is sampled from it using the gumbel-softmax.

*Edge construction* Previous work has shown that text plays the most important role in MAS, so we construct edges centered around text. As shown in Fig. 3, firstly, we employ a fully connected solution to link the nodes, which from the same modality. And then, for nodes from different modality, we connect these nodes according to audio—to—text and video—to—text standards. After the above operation, we can obtain the local graph GL = (AL, VL), where AL represents the adjacency matrix and VL is the node feature.

#### Building global graph

To leverage the intricate sentiment implications within local features, we construct a local multimodal diagram based on the original sequence features.

We obtain the codebook ${CB}_{m}$, m $\in${t, a, v} for each modality in section “Create codebook”, which is a two-dimensional matrix containing the global co-occurrence features of the dataset. Therefore, for each utterance, we use the corresponding codebook to map the sequence features of each modality. Same as in Sect. 3.2.1, we explain this mapping process using acoustic modalities.

3 $$X_{a}^{\prime } = \left\{ {CB_{a}^{{id_{i} }} } \right.\left| {i = 1, \ldots ,\left. {T_{a} } \right\}} \right. \in {\mathbb{R}}^{{T_{a} \times d_{a} }}$$

where

4 $$id_{i} = \arg \,\min \left\| {CB_{a}^{k} - } \right.\left. {x_{a}^{i} } \right\|,\quad k = 1, \ldots ,K_{a}$$

where $X_{a}^{\prime }$ is the global acoustic sequence features, ${CB}_{a}^{i{d}_{i}}$ represents the *i* ${d}_{i}$-th vector of C ${B}_{a}$, *i* ${d}_{i}$ represents the index value of ai after mapping by C ${B}_{a}$. The same operation is applied to the original sequence features of text and video, the same operation is applied to the raw sequence features of text and video, and obtain textual global sequence features $X_{t}^{\prime }$ and visual global sequence features $X_{v}^{\prime }$. Finally, we use the same approach as in section “Building local graph” to construct a global multimodal graph ${G}_{G}$ = (${A}_{G}$, ${V}_{G}$), where ${A}_{G}$ represents the adjacency matrix and ${V}_{G}$ is the node feature, to explore global level information interactions.

### Hierarchical graph contrastive learning

This section consists of four parts local-level graph contrastive learning, global-level graph contrastive and cross-level graph contrastive learning and fusion and sentiment prediction. The following sections discuss the details of the three parts.

#### Local-level graph contrastive learning

In order to explore local information representation in multimodal emotion extraction, we design the local-level graph contrastive learning. Firstly, given a local graph ${G}_{L}$ = (${A}_{L}$, ${V}_{L}$), an automatic graph augmentation strategy (section “Hierarchical graph contrastive learning”) is used to obtain the augmented graph ${G}_{L}^{\prime}$ = (${A}_{L}$, ${V}_{L}$). And then, the graph encoder (section “Automatic graph data augmentation strategy”) takes ${G}_{L}$ and ${G}_{L}^{\prime}$ as input and outputs latent representation of ${G}_{L}$ and ${G}_{L}^{\prime}$.

5 $$H_{L} = GraphEnconder(G_{L} )$$

6 $$H_{L}^{\prime } = GraphEncoder(G_{L}^{\prime } )$$

where ${H}_{L}$ and $H_{L}^{\prime }$ denote the latent semantic features of ${G}_{L}$ and $G_{L}^{\prime }$, respectively. We expect the representations also hold the invariance property held by the final outputs. To do so, we separately consider the encoder and decoder in the graph neural network. Following the theory of Ji et al.^23^. For the encoder, we introduce the readout function, which is global mean pooling, to consider the invariance property at the graph level.

7 $$z_{L} = READOUT(H_{L} )$$

8 $$z_{L}^{\prime } = READOUT(H_{L}^{\prime } )$$

where READOUT(**∙**) is the readout function, ${z}_{L}$ and ${z}_{L}^{\prime}$ represents the global of ${H}_{L}$ and ${H}_{L}^{\prime}$. And for decoder, we employ fully-connected layers as decoder to keep invariance property at the node level.

9 $$G_{(L,r)} = Deconder(H_{L} ) = \left( {A_{L} ,V_{(L,r)} } \right)$$

Based on it, given N examples in a mini-batch, we design a loss function for local level graph contrastive learning:

10 $$Loss_{local} = Loss_{(L,n)} + \alpha L_{(L,g)}$$

11 $$Loss_{(L,n)} = \frac{1}{N}{{\sum\limits_{i = 1}^{N} {\left\| {V_{L}^{i} - \left. {V_{(L,r)}^{i} } \right\|} \right.}^{2} } \mathord{\left/ {\vphantom {{\sum\limits_{i = 1}^{N} {\left\| {V_{L}^{i} - \left. {V_{(L,r)}^{i} } \right\|} \right.}^{2} } {\left| {V_{L}^{i} } \right|}}} \right. \kern-0pt} {\left| {V_{L}^{i} } \right|}}$$

12 $$Loss_{(L,g)} = \frac{1}{N}\sum\limits_{i = 1}^{N} {\left\| {z_{L}^{i} - z_{L}^{^{\prime}i} } \right\|}$$

where ${Loss}_{(L,n)}$ and ${Loss}_{(L,g)}$ represent the comparative loss at the node- and graph-level self-supervised contrastive loss, respectively. The superscript i denotes the index value of the mini-batch, $\left|{V}_{L}^{i}\right|$ deontes the number of nodes in the *i*-th graph, α is the hyperparameter that adjusts the balance.

#### Cross-level graph contrastive learning

From local- and global- level graph contrastive learning we can obtain the local- and global-latent graph representations. They are different potential representations from the same sample, which refer to the same sentiment information. Cross-Level Graph Contrastive Learning aim to learn two encoders such that embeddings in two modalities are close to each other in the learned space. There, we define *H*_*L*_ and *H*_*G*_ as a positive sample pair. We apply nonlinear projection *MLP* with shared parameters to convert embeddings from different representations to the same space for comparison.

16 $$p_{L} = MLP(H_{L} )$$

17 $$p_{G} = MLP(H_{G} )$$

The contrastive loss in cross-level Graph Contrastive Learning is formulated as:

18 $$Loss_{cross} = - \log \sum\limits_{i = 1}^{N} {\frac{{\exp \left[ {sim\left( {p_{L}^{i} ,p_{G}^{j} } \right)/\tau } \right]}}{{\sum {_{j = 1}^{N} \exp \left[ {sim\left( {p_{L}^{i} ,p_{G}^{j} } \right)/\tau } \right]} }}}$$

where sim (**∙**) is the cosine similarity, τ is the temperature value.

#### Fusion and sentiment prediction

The concatenation of two representation is regarded as the fusion results and is fed into a simple classifier to make a final prediction of the sentiment intensity.

19 $$O = Concat\left[ {z_{L} \left\| {z_{G} } \right.} \right]$$

20 $$\hat{y} = W_{1} \cdot {\text{LeakReLU}} \left( {W_{2} \cdot {\text{BN}} (O) + b_{2} } \right) + b_{1}$$

where BN is the BatchNorm operation, and LeakyReLu is used as activation.

#### Model training

Along with the graph contrastive learning loss the overall learning of the model is performed by minimizing:

21 $$L = \frac{1}{N}\sum\limits_{i}^{N} {\left( {\left| {\hat{y}_{i} } \right.\left. { - y_{i} } \right|} \right)} + \beta Loss_{cross} + \gamma \left( {Loss_{local} + Lossg_{lobal} } \right)$$

where $\widehat{y}$ is predict output of model and the* y* is true label, *β* and *γ* are hyperparameter, controlling the effect of different losses.

### Automatic graph data augmentation strategy

To better explore the structure of graphs, inspired by^29^, we introduce an automatic graph data augmentation model.

#### Framework of Automatic Graph Data Augmentation

As shown in Fig. 3, Given a graph G We use GIN^30^ layers to get the node embedding from the node attribute.

22 $$h_{v}^{(n)} = GIN^{{({\text{n}})}} \left( {h_{v}^{(n - 1)} } \right)$$

We use *n* GIN layers as the embedding layer, we denote ${h}_{v}^{(n)}$ as the embedding of node *v* after the *n*-th layer.

For each node, we use the embedded node feature to predict the probability of selecting a certain augment operation. The augmentation pool for each node is drop, keep, and mean-mask. We employ the gumbel-softamx^30^ to sample from these probabilities then assign an augmentation operation to each node.

23 $$f_{v} = {\text{GumbelSoftmax}} \left( {h_{v}^{(n)} } \right)$$

For node *v*, we have the node feature *x*_*v*_, the augmentation choice *fv*, and the function Aug(*x, f*) for applying the augmentation. Then the augmented feature $x_{v}^{\prime }$ of node *v* is obtained via:

24 $$x_{v}^{\prime } = Aug(x_{v} ,f_{v} )$$

The dimension of the last layer n is set as the same number of possible augmentations for each node. Therefore, ${h}_{v}^{(n)}$ denotes the probability distribution for selecting each kind of augmentation. *f*_*v*_ is a one-hot vector sampled from this distribution via gumbel-softmax.

#### Training of automatic graph data augmentation

**Figure 4:**
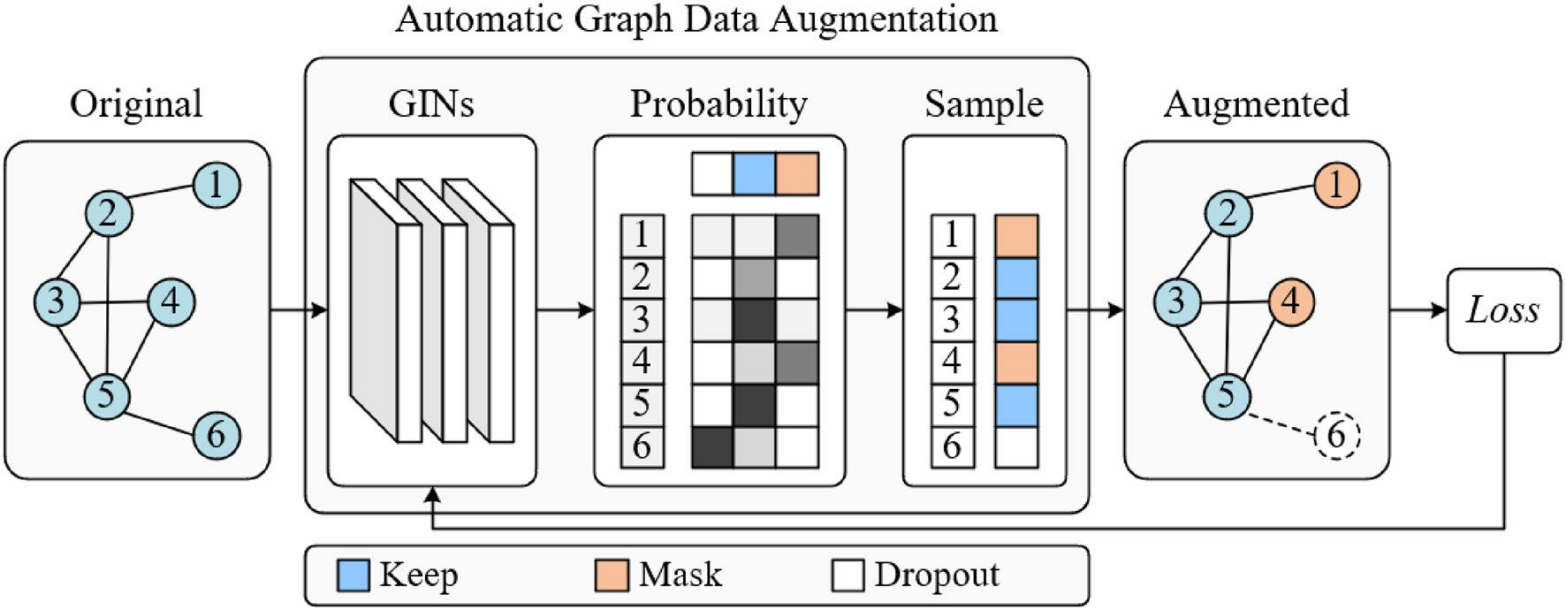
Training of automatic graph data augmentation.

According to InfoMin principle^31^, a good positive sample pair for contrastive learning should maximize the label-related information as well as minimize the mutual information (edge similarity) between them. Base on it, we designed a training process (see Fig. 4).For the label-related information, firstly, we use the graph encoder (section “Fusion and sentiment prediction”) to fuse information between nodes.

25 $$H_{raw} = GraphEncoder(G_{raw} )$$

26 $$H_{aug} = GraphEncoder(G_{aug} )$$

where *G*_*raw*_ and *G*_*aug*_ denote the raw graph and augmented graph, H and H′ denote Corresponding node features after encoder. And then, global mean pooling is used to obtain a graph-level representation (z_raw_ and z_aug_) of each graph. Next, z and z′ are fed into two feedforward neural networks to obtain the predicted sentiment scores.

27 $$\hat{y}_{raw} = W_{4} (W_{3} {\text{z}}_{{{\text{raw}}}} {\text{ + b}}_{{3}} ) + b_{4}$$

28 $$\hat{y}_{aug} = W_{6} (W_{5} {\text{z}}_{{{\text{aug}}}} {\text{ + b}}_{{5}} ) + b_{6}$$

where *W*_3_, *W*_4_, *W*_5_, *W*_6_ represents the learnable weight, *b*_3_, *b*_4_, *b*_5_, *b*_6_ represents the learnable bias. We directly use the mean absolute error (MAE) loss, the loss function is calculated as follows:

29 $$L_{mae} = \frac{1}{N}\sum\limits_{i = 1}^{N} {\left[ {\left| {\hat{y}_{raw}^{i} } \right. - \left. {y^{i} } \right| + \left| {\hat{y}_{aug}^{i} } \right. - \left. {y^{i} } \right|} \right]}$$

For mutual information, during the view generation process, we have a sampled state matrix S indicting each node’s corresponding augmentation operation. For a graph *G*, we denote the sampled augmentation choice matrix as *A*_1_ and define a sampling state matrix with all ‘keep’ as *A*_*2*_, then we formulate the similarity loss *L*_*sim*_ as:

30 $$L_{sim} = sim\left( {A_{1} ,A_{2} } \right)$$

where sim (a, b) denotes the cosine similarity between A and B. the overall learning of the model is performed by minimizing:

31 $$Loss = L_{mae} + L_{sim}$$

### Graph representation learning

Based on our graph structure, we employ Graph Attention Network^20^ to update the nodes in the graphs by aggregating the information from the neighborhoods with varying weights. Specifically, for the current node *v*_*i*_ and the neighbor node *v*_*j*_, concatenating them and then mapping to a scalar *s*_*ij*_ as the attention coefficient.

32 $$s_{ij} = Leaky\,{\text{Re}} {\text{LU}} \left( {a\left[ {Wh_{i} \left\| {Wh_{j} } \right.} \right]} \right)$$

where *a* is a weight vector, *W* is a weight matrix, and ‖ is the concatenation operation. Then normalizing the attention coefficients of all neighbors by softmax.

33 $$a_{ij} = soft\,\max_{j} (s_{ij} ) = \frac{{\exp (s_{ij} )}}{{\sum\nolimits_{k \in N} {\exp (s_{ik} )} }}$$

where *N*_*i*_ denotes the set of node *i* and its neighbors. Finally, the representation of node *i* is updated with a weighted sum of the representations of neighbors and itself, and multi-head attention mechanism is applied to stabilize the learning process of self-attention.

34 $$\tilde{h}_{i} = \left\| {_{k = 1}^{k} \left( {\sum\limits_{j \in N} {a_{ij}^{k} W^{k} h_{j} } } \right)} \right.$$

where k denotes the *k*-th attention head.

35 $$L = \frac{1}{N}\sum\limits_{i}^{N} {\left( {\left| {\hat{y} - y} \right|} \right)} + \alpha L_{cross} + \beta \left( {L_{local} + L_{global} } \right)$$

where $\widehat{y}$ is predict output of model and the* y* is true label, *α*, *β* and *γ* are hyperparameter, controlling the effect of different losses.

## Experiments

The experiment was conducted on a high-performance computing cluster consisting of four NVIDIA GeForce RTX 3090 GPUs, which provided immense computational power. The cluster was interconnected with high-speed networking to ensure efficient data communication and parallel processing.

### Experiment settings

#### Datasets

In this work, experiments are conducted on two public multimodal sentiment analysis datasets, CMU-MOSI^32^ and CMU-MOSEI^33^. The basic statistics of each dataset are shown in Table 1. Here, we give a brief introduction to the above datasets.

**Table 1:** Dataset basic statistics for benchmark MSA dataset.

Dataset	#Train	#Test	#Valid	#All
MOSI	1283	229	686	2198
MOSEI	16,326	1871	4659	22,856

*CMU-MOSI* The CMU-MOSI dataset is one of the most popular benchmark datasets for MSA. The dataset contains 2199 short monologue video clips taken from 93 YouTube movie review videos. The utterances are manually annotated with a sentiment score from − 3 (strongly negative) to 3 (strongly positive).

*CMU-MOSEI* CMU-MOSEI is enlarged from the CMU-MOSI. It has the same annotations as the CMU-MOSI. In CMU-MOSEI, there are 16,326 utterances for training, 1871 utterances for validation, and 4659 utterances for testing.

#### Evaluation metrics

For a comprehensive comparison with baselines, we use public evaluation metrics of classification and regression to demonstrate the performance of our proposed framework and further compare with baselines: seven-class classification accuracy (Acc7) indicating the correct sentiment label predictions in the range of [− 3, + 3], binary classification (Acc2) and F1-score, mean absolute error (MAE) computing the average absolute difference between predicted and truth labels, Pearson correlation (Corr) measuring the degree of prediction skew.

#### Implementation details

The results of our model take the average results obtained from five runs with different random seeds for obtaining stable results. Detailed training settings are presented in Table 2. In addition, we use a learning rate adjustment strategy to update the learning rate when training. Among them, *α*, *β* and *γ* are the most suitable values that we find by using the grid search.

**Table 2:** Training setting details.

Parameter	MOSI		MOSEI	
	Aligned	Unaligned	Aligned	Unaligned
Epoch	30	30	15	15
Batch size	64	8	64	8
GAT layers	3	4	3	4
GAT heads	4	4	4	4
HGGL-LG LR	5e−4	1e−4	5e−4	1e−4
Other LR	1e−3	1e−3	1e−3	1e−3
Dropout	0.3	0.3	0.3	0.3
α	0.1	0.1	0.1	0.1
β	0.01	0.01	0.01	0.01
γ	0.1	0.1	0.1	0.1

*LR* learning rate.

### Baselines

*LMF*^8^ Low-rank Multimodal Fusion (LMF) is a method that leveraging low-rank weight tensors to make multimodal fusion efficient without compromising on performance. It not only drastically reduces computational complexity but also significantly improves performance.But it still has some disadvantages, such as high computational resource requirements, weak ability to handle noise and redundancy, and susceptibility to interference.

*TFN* Tensor Fusion Network (TFN)^9^ utilizes tensor fusion layer where a cartesian product is used to form a feature vector. Therefore, information from three modalities can be fused to predict the sentiment.The main disadvantages of TFN include high computational complexity, sensitivity to noise and outliers, dependency on parameters and model structure, limited interpretability, and the need for a large amount of annotated data.

*MISA* By projecting each modality of samples into two subspaces, this method learns both modality-invariant and -specific representations^13^, which then are fused for sentiment analysis.

*MulT* Multimodal Transformer^21^ extends three sets of Transformers with directional pairwise cross-modal attention which latently adapts streams from one modality to another.During use, special attention should be paid to the limitations of cross-modal attention mechanisms and the complexity of deployment and configuration.

*Self-MM*^2^ Self-Supervised Multi-Task Learning automatically generates unimodal labels which are weight-adjusted by multimodal labels to learn consistency and difference across modalities. The disadvantages of the Self-MM model include high computational complexity, large data requirements, challenges in modality alignment, limited generalization ability, and limited interpretability.

*TCM-LSTM*^34^ Learn inter-modality dynamics in a different perspective via acoustic- and visual- LSTMs where language features play dominant role.The disadvantages of the TCM-LSTM model include high computational complexity, challenges in parameter adjustment, sensitivity to initial states, tendency to local optima, and vulnerability to noise and outliers.

*MTAG*^15^ Modal-Temporal Attention Graph (MTAG) can capable of both fusion and alignment. while utilizing substantially lower number of parameters than a transformer-based model such as MulT^33^.The disadvantages of the MTAG model include high computational complexity, long training time, sensitivity to noise and outliers, challenges in parameter adjustment, and difficulty in handling large-scale graph data.

*GraphCAGE*^35^ Graph Capsule Aggregation (GraphCAGE) to model unaligned multimodal sequences with graph-based neural model and Capsule Network.The disadvantages of GraphCAGE include high computational complexity, stringent requirements on data quality and scale, and the need for extensive labeled data.

### Comparison with baseline

We evaluate the HGCL-LG model on the CMU-MOSI dataset, Table 3 shows the experiment results. From the results, we observe that HGCL-LG outperforms all the baseline models on the two datasets in most cases, which verifies the effectiveness of our approach in the MSA task. This indicates that exploring the sentiment implications from both local- and global levels is significant for improving the performance of MSA. Through T-test analysis, we found significant differences in the average values between the two groups of data (*p* < 0.05). This indicates that the method has significant test results on CMU-MOSI and CMU-MOSEI. Moreover, our proposed model works well on both aligned and unaligned datasets, but since we do not explicitly model the aligned data, the results on unaligned datasets are slightly worse than on aligned datasets, our proposed model works well on both aligned and unaligned datasets, but since we do not explicitly model the aligned data, the results on unaligned datasets are slightly worse than on aligned datasets.

**Table 3:** Main results on MOSI and MOSEI.

Models	CMU-MOSI					CMU-MOSEI					Data setting
	Acc7↑	Acc2↑	F1↑	MAE↓	Corr↑	Acc7↑	Acc2↑	F1↑	MAE↓	Corr↑	
TFN*^8^	33.7	78.3	78.2	*0.925*^a^	0.662	**52.2**^u^	81.0	81.1	0.570	0.716	u
LMF*^9^	32.7	77.5	77.3	0.931	0.670	*52.0*^u^	81.3	81.6	*0.568*^u^	*0.727*^u^	u
MuLT^†^^20^	35.5	80.6	79.3	0.972	0.681	49.0	81.4	81.7	0.630	0.664	a
MISA^‡^^13^	*43.5*^a^	81.8	81.7	0.752	*0.784*^a^	**52.2**^a^	81.6	82.0	0.550	*0.758*^a^	a
Self-MM*^2^	**45.8**^a^	*82.7*^a^	*82.6*^a^	*0.731*^a^	0.731	50.6	*82.6*^a^	*82.8*^a^	*0.547*^a^	0.752	a
TCM-LSTM^†^^34^	35.4	81.7	81.8	0.903	0.672	50.6	81.4	81.6	0.606	0.673	a
MTAG^†^^14^	31.9	80.5	80.4	0.941	*0.692*^u^	48.2	79.1	75.9	0.645	0.614	u
GraphCAGE^35^	**35.4**^u^	*82.1*^u^	*82.1*^u^	0.933	0.684	48.9	*81.7*^u^	*81.8*^u^	0.609	0.670	u
HGG-LG(our)	*41.7*^a^	**84.0**^a^	**83.9**^a^	**0.725**^a^	**0.788**^a^	*49.3*^a^	**84.2**^a^	**84.3**^a^	**0.545**^a^	**0.769**^a^	a
HGG-LG(our)	*35.1*^u^	**83.5**^u^	**83.6**^u^	**0.765**^u^	**0.776**^u^	49.5	**84.0**^u^	**84.1**^u^	**0.511**^u^	**0.753**^u^	u

↑ denotes the higher the evaluation metric the better, and ↓ denotes the lower the evaluation metric the better. Result * represents the results we achieved in the laboratory, where Self-MM * is reproduced using the source code released by the authors. Result with † indicate the result from^4^, and with ‡ presents the result from^2^, For data setting, a and u represent aligned and unaligned, respectively. The bold represents the best result, and the italic is the second-best result.

In general, the hierarchical graph contrast learning proposed by us can fully learn the local information and global co-occurrence features of samples, which can significantly improve the precision of MSA tasks.

### Ablation study

To verify the impact of the hierarchical graph contrastive learning on performance, we conduct ablation experiments on the two datasets and show the results in Table 4. From Table 4, we can see that the removal of any module in HGCL-LG results in a decline in model performance. For contrastive learning (CL), the result demonstrates that *L*_*c*_ and *L*_*l&g*_ designed by us can well explore the global information and local information of multi-modal instances, and enable the model to learn the complex relationship between local information and global information. For edge types, “V → T ← A” is the most effective edge construction method, this indicates that the other two methods produce negative noise characteristics in message aggregation. Then, for information types, bath local features and global features play an important role in MSA tasks. Finally, we evaluate the validity of global contribution characteristics, “CMU-MOSI” means using CMU-MOSI codebook to build the global graph of CMU-MOSI, “CMU-MOSEI” means using CMU-MOSEI codebook to build the global graph of CMU-MOSI, the results show that the extracted global co-occurrence feature can effectively represent emotion information.

**Table 4:** Ablation studies on aligned CMU-MOSI validation dataset.

Ablation	Acc2↑	F1↑	MAE↓	Corr↑
Contrastive learning(CL)				
*L*_*c*_, *L*_*l&g*_	**84.0**	**83.9**	**0.725**	**0.788**
*L*_*c*_	82.5	82.6	0.736	0.778
*L*_*l&g*_	83.1	83.0	0.730	0.780
Edge types(Fig. 2)				
V → T ← A	**84.0**	**83.9**	**0.725**	**0.788**
T ← A ← V	83.1	83.0	0.736	0.742
T ← V ← A	82.3	82.1	0.749	0.727
Information types (No CL)				
Local only	82.1	82.2	0.712	0.720
Global only	81.5	81.5	0.722	0.717
Local, global	**84.0**	**83.9**	**0.725**	**0.788**
Codebook				
CMU-MOSI	**84.0**	**83.9**	**0.725**	**0.788**
CMU-MOSEI	83.5	83.6	0.730	0.780

Best results are highlighted in bold. *L*_*c*_ denotes the cross-level graph contrastive loss, and *L*_*l&g*_ represents the sum of *L*_*local*_ and *L*_*global*_*.*

### Representation visualization

**Figure 5:**
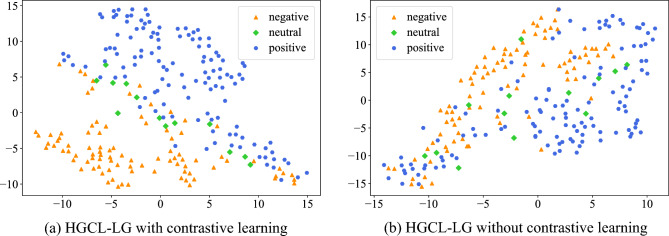
T-SNE^36^ visualization of multimodal representation in the embedding space on the valid set of CMU-MOSI.

Figure 5 displays the visualization of fusion multimodal representation ***O*** calculated by HGCL-LG with contrastive learning losses or not. Without contrastive learning, the representation of positive and negative samples is highly distinguishable, but neutral samples are distributed discretely, which means that the model does not learn the relationship between the local information of the sample and the global co-occurrence feature. After introducing designed contrastive learning, the positive and negative samples have a clearer dividing line, and the neutral samples are distributed along the dividing line. This shows that contrastive learning can effectively improve the discrimination of the model to different samples, which also proves the effectiveness of the designed contrastive learning tasks on representation learning.

### Case study

**Figure 6:**
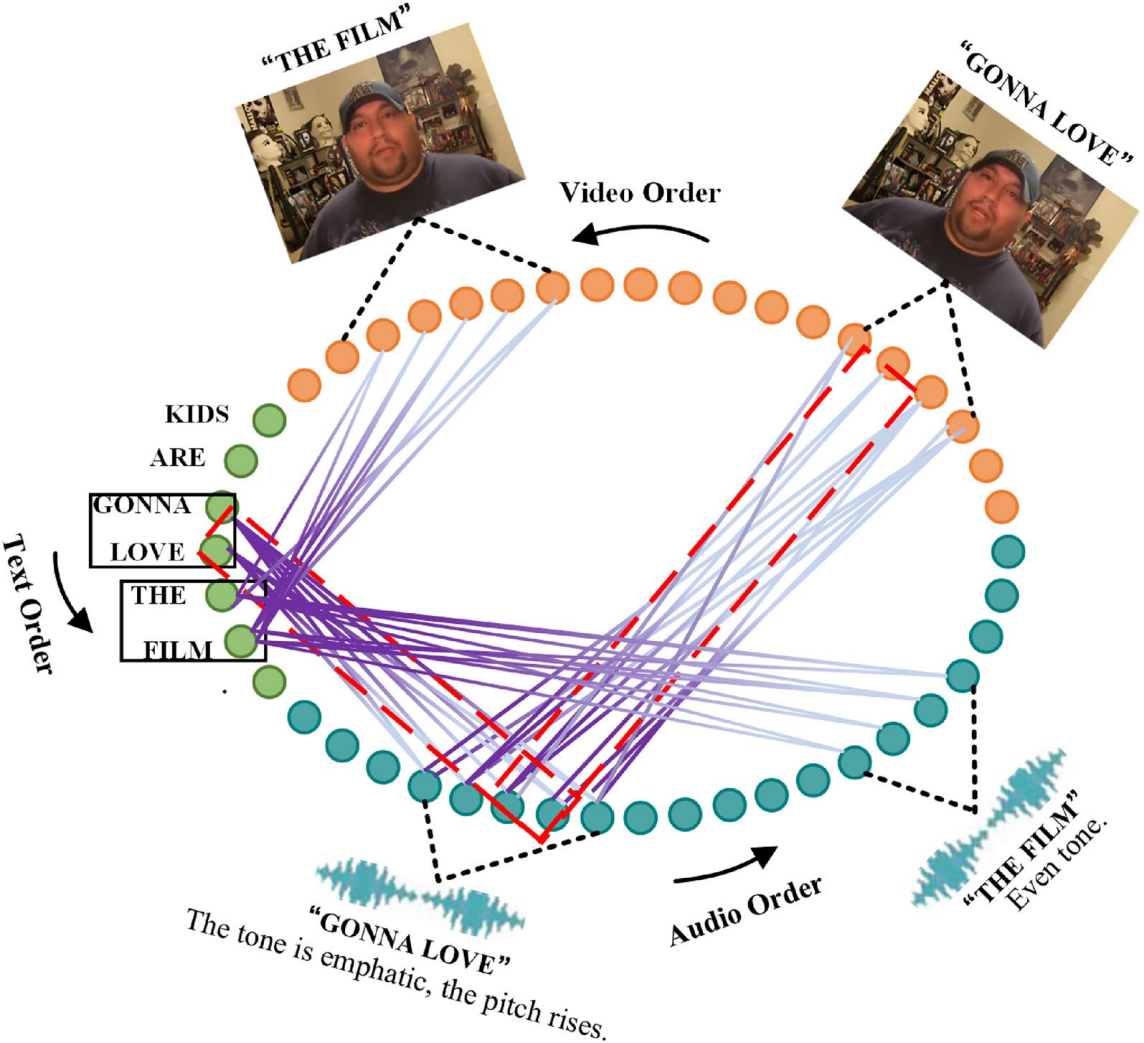
Case study on the application of Graph Neural Networks in Multimodal Sentiment Analysis (The image is from CMU-MOSI^32^. The dataset is publicly available for download).

We show in Fig. 6 a case study on the application of Graph Neural Networks in Multimodal Sentiment Analysis (The image is from CMU-MOSI^32^. The dataset is publicly available for download with all the extracted features^32^). First, the non-aligned multimodal sequences are transformed into a graph with heterogeneous nodes and edges, which can capture interactions between different modalities over time. Then, this graph is effectively processed using multimodal temporal attention. The sentiment analysis results are obtained by detection on popular models.The method has been recognized by relevant workers, demonstrating the applicability of Graph Neural Network models in the real world.

## Conclusion

This paper proposes a novel hierarchical graph contrastive learning (HGCL-LG) framework for multimodal sentiment analysis (MSA), in which graph contrastive learning is performed at local-level, global-level and cross-level. For the graph contrastive learning strategy performed at local-level and global-level, we devise a node-based contrastive loss and a graph-based contrastive loss.The node-based contrastive loss is devised to improve the learning of sentiment cues by capturing the latent sentiment representation of the local/global graph. And the cross-level contrastive loss is devised to make use of sentiment relations within local graph and global graph. In addition, in order to explore better multi-modal graph structures, we introduce an adaptive graph augmentation mechanism for automatic graph augmentation. Experimental results on two benchmark datasets show that our method outperforms the state-of-the-art baselines in MSA.

## Data Availability

All data generated or analysed during this study are included in this published article. Statement: The images of the subjects in Fig. 6 are from CMU-MOSI, and all data in this dataset can be downloaded publicly. All subjects and/or their legal guardians have agreed to publish their identifying information or images in Scientific Reports after being fully informed.
